# High Rate Performance Nanocomposite Electrode of Mesoporous Manganese Dioxide/Silver Nanowires in KI Electrolytes

**DOI:** 10.3390/nano5041638

**Published:** 2015-10-13

**Authors:** Yanhua Jiang, Xiuguo Cui, Lei Zu, Zhongkai Hu, Jing Gan, Huiqin Lian, Yang Liu, Guangjian Xing

**Affiliations:** 1 College of Materials Science & Engineering, Beijing Institute of Petrochemical Technology, Beijing 102617, China; E-Mails: jiangyanhua@bipt.edu.cn (Y.J.); zulei@buct.edu.cn (L.Z.); huzhongkai@bipt.edu.cn (Z.H.); ganjing@bipt.edu.cn (J.G.); yang.liu@bipt.edu.cn (Y.L.); xingguangjian@bipt.edu.cn (G.X.); 2 State Key Laboratory of Organic-Inorganic Composites, Beijing University of Chemical Technology, Beijing 100029, China; 3 Beijing Key Laboratory of Specialty Elastomer Composite Materials, Beijing Institute of Petrochemical Technology, Beijing 102617, China

**Keywords:** mesoporous manganese dioxide, silver nanowires, electrolyte, specific capacitance

## Abstract

In recent years, manganese dioxide has become a research hotspot as an electrode material because of its low price. However, it has also become an obstacle to industrialization due to its low ratio of capacitance and the low rate performance which is caused by the poor electrical conductivity. In this study, a KI solution with electrochemical activity was innovatively applied to the electrolyte, and we systematically investigated the rate performance of the mesoporous manganese dioxide and the composite electrode with silver nanowires in supercapacitors. The results showed that when mesoporous manganese dioxide and mesoporous manganese dioxide/silver nanowires composite were used as electrodes, the strength of the current was amplified five times (from 0.1 to 0.5 A/g), the remaining rates of specific capacitance were 95% (from 205.5 down to 197.1 F/g) and 92% (from 208.1 down to 191.7 F/g) in the KI electrolyte, and the rate performance was much higher than which in an Na_2_SO_4_ electrolyte with a remaining rate of 25% (from 200.3 down to 49.1 F/g) and 60% (from 187.2 down to 113.1 F/g). The morphology and detail structure were investigated by Scanning electron microscopy, X-ray diffraction, Fourier transform infrared spectrometry and Nitrogen adsorption-desorption isotherms. The electrochemical performance was assessed by cyclic voltammograms, galvanostatic charge/discharge and electrochemical impedance spectroscopy.

## 1. Introduction

In recent years, supercapacitors, also known as electrochemical capacitors, have become very popular as a new type of energy storage device due to their exceptional properties such as fast charge/discharge capacity, high power density, long life cycle, eco-friendliness and safety [1]. With these merits, supercapacitors have become very competitive for applications in areas such as electric hybrid vehicles, digital communication devices such as mobile phones, digital cameras, electrical tools, pulse laser techniques, and storage of energy generated by solar cells [2]. Therefore, supercapacitors perfectly fill the gap between traditional batteries and conventional capacitors [3,4]. There are two major types of supercapacitors, as categorized by their energy storage mechanism: electric double layer capacitors, and Faraday pseudo capacitors. Faraday pseudo capacitors employ Faradaic reactions occurring at the very nearly electrode interface and use electroactive materials to store energy through redox reactions [5,6].

The electrode material is an important factor to directly decide the capability, delivery rates and efficiency of supercapacitors. Several transition metal oxide materials could act as effective electrode materials for supercapacitors, including ruthenium oxide, manganese dioxide (MnO_2_), nickel oxide, and nickel hydroxide, among others [7]. However, some of the most capable materials are prohibitively expensive for use in commercial applications, such as ruthenium oxide and cobalt hydroxide. Recently, many researchers have focused on some cheaper metal oxides materials, such as manganese dioxide and nickel oxide, *etc*. [8,9]. The MnO_2_ has been widely studied as a supercapacitor electrode material due to its good environmental stability, high potential storage capacities and high potential window. However, due to its low electrical and ionic conductivity, its use in supercapacitors has been limited [10]. In order to overcome these limitations, mesoporous MnO_2_ could be prepared in a way that results in high specific surface area. Indeed, some papers have suggested that MnO_2_ can be combined with other highly conductive materials, such as graphene [11,12,13,14], mesoporous carbon [15,16], polyaniline [17,18,19] and polypyrrole [20,21] to improve the electrical conductivity. Otherwise, there are only a few previous reports related to the application of nanoporous metal/manganese dioxide based materials for supercapacitors [22].

The specific surface area of electrode materials is an important factor that can affect the electrochemical performance of supercapacitor. A larger specific surface area on an electrode will provide more electroactive sites to improve the electrochemical performance. Therefore, mesoporous MnO_2_ with a large specific surface area will show a better electrochemical performance than bulk. Conductivity of mesoporous MnO_2_ can also be increased by the addition of conductive metals. Apart from the electrode materials, the electrolyte is also particularly important in affecting the performance of supercapacitors. Some papers published in recent years have proposed that the total capacitance and energy density of supercapacitors would be greatly improved if an electrochemically-active material were added into the electrolyte [23,24].

In this study, a KI solution with electrochemical activity was innovatively applied into an electrolyte. Subsequently, the rate performance of the mesoporous manganese dioxide and the composite electrode with silver nanowires in supercapacitors was systematically investigated. The results showed that when mesoporous MnO_2_ and mesoporous MnO_2_/silver nanowires were composited as an electrode, the strength of the current was amplified 5 times (from 0.1 to 0.5 A/g), the remain rates of specific capacitance were 95% and 92% in the KI electrolyte, and the rate performance were much higher than a Na_2_SO_4_ electrolyte, where the remain rate were 25% and 60%. Results indicate that KI electrolytes may become a new mode for supercapacitors to change their specific capacitance.

## 2. Results and Discussion

### 2.1. Effect of the Amount of Acid on the Structure of MnO_2_

Because potassium permanganate is a strong oxidizing agent and ethylene glycol is a strong reducing agent, the exothermic redox reaction between the two of them leads to the formation of MnO_2_ and other oxidized products, such as aldehydes or a mixture of aldehydes, carboxylic acids and even CO_2_. The formation process may be explained as follows: Once the ethylene glycol was added into the KMnO_4_ acid solution, MnO_2_ nuclei were formed immediately, which acted as the precursor. Concentrated sulfuric acid was then added in the solution, increasing the oxidizability of the potassium permanganate and further increasing the reaction rate. On the other hand, acid can also “etch” the originally produced MnO_2_, thereby increase the specific surface area. However, adding an excess of acid could not increase of surface area correspondingly, because if the acid concentration is too high, the oxidation of potassium permanganate would become stronger and the reaction rate was further increased, the originally produced MnO_2_ nuclei was too late to be etched, so the regeneration ability also cannot continue [25].

**Figure 1:**
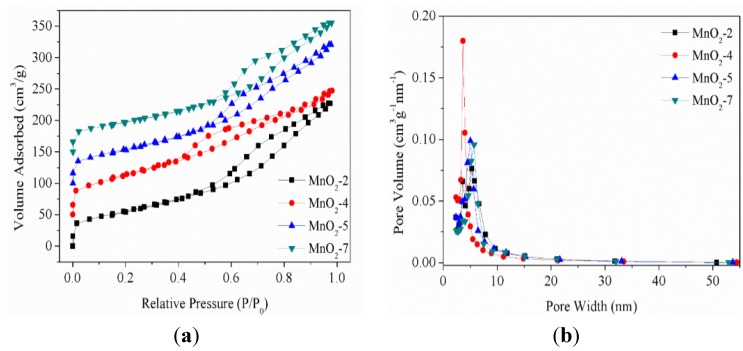
Nitrogen adsorption/desorption isotherms and BJH pore-size distribution curves of the samples MnO_2_-2, MnO_2_-4, MnO_2_-5, MnO_2_-7. (**a**) Nitrogen adsorption/desorption isotherms; (**b**) Pore-size distribution curves.

The samples of MnO_2_-2, MnO_2_-4, MnO_2_-5, MnO_2_-7 were prepared by adding 2, 4, 5 and 7 mL of concentrated sulfuric acid into the reaction conditions, respectively. The BET surface areas of the samples were 204.41, 232.96, 203.54 and 175.57 m^2^/g, respectively, and the pore diameters of the samples were 5.31, 3.64, 3.90 and 5.71 nm, respectively. As can be seen in Figure 1, all of the isotherms were type IV and displayed hysteresis loops in the relative pressure range of 0.6–0.9. Furthermore, a plateau appeared in the adsorption branch, suggesting that all of the samples were mesoporous. However, the nitrogen sorption isotherm of MnO_2_-4 exhibited a hysteresis loop in the relative pressure range of 0.4–0.7 and the pore diameter was the smallest among all samples. This conclusion indicates that the mesoporous structure of MnO_2_-4 was more uniform than the others, so the MnO_2_-4 was used for the preparation of the composite materials.

### 2.2. Microstructure Characterization

**Figure 2:**
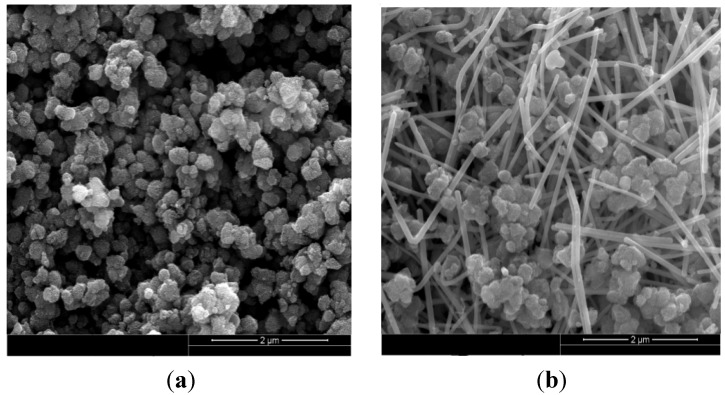
Scanning electron microscopy (SEM) images of mesoporous MnO_2_, mesoporous MnO_2_/silver nanowires. (**a**) Mesoporous MnO_2_; (**b**) Mesoporous MnO_2_/silver nanowires.

Figure 2 shows the scanning electron microscopy (SEM) images of mesoporous MnO_2_ and mesoporous MnO_2_/silver nanowires nanocomposite. As shown in Figure 2a, the mesoporous MnO_2_ has a uniform particle morphology. And in Figure 2b, the mesoporous MnO_2_ and the silver nanowires have dispersed uniformly, which provide a chance to improve the electrochemical performance of supercapacitors.

**Figure 3:**
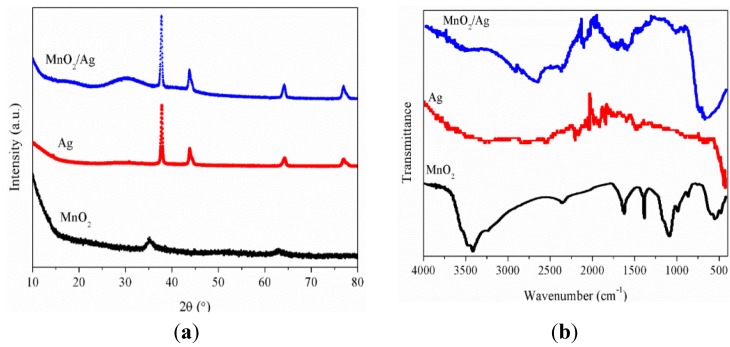
(**a**) X-ray diffraction (XRD) spectra of mesoporous MnO_2_, silver nanowires and mesoporous MnO_2_/silver nanowires; (**b**) Fourier transform infrared spectrometry (FT-IR) spectra of mesoporous MnO_2_, silver nanowires and mesoporous MnO_2_/silver nanowires.

Figure 3a depicted the X-ray diffraction (XRD) pattern of the as-prepared mesoporous MnO_2_, silver nanowires and mesoporous MnO_2_/silver nanowires. As shown in Figure 3a, the main diffraction peaks at 2θ = 35.6° and 63.8° in the as-prepared mesoporous MnO_2_, the diffraction angles at 38.2°, 44.5°, 64.5° and 77.2° in the as-prepared silver nanowires, all the reflections can be indexed to the face centered cubic structure according to the previous published papers [26,27]. In contrast with the as-prepared silver nanowires, the diffraction peaks at 38.2°, 44.5°, 64.5° and 77.2° also existed in the mesoporous MnO_2_/silver nanowires; moreover, the diffraction angles at 35.6° which respected the mesoporous MnO_2_ crystalline phase caused a slight shift to occur, and set the diffraction angles at 30.1°.

To obtain more specific information about the as-prepared mesoporous MnO_2_/silver nanowires, Fourier transform infrared spectrometry (FT-IR) spectra of the mesoporous MnO_2_, silver nanowires and the composite were obtained. As seen from Figure 3b, the spectrum of MnO_2_ shows a broad absorption band at 3424 cm^−1^, which can be assigned to the O–H stretching vibration, and the peak at 532 cm^−1^ should be ascribed to the contribution of the Mn–O vibrations, while other three pronounced peaks centered at 1081, 1384 and 1620 cm^−1^ are O–H stretching vibrations on Mn atom. In contrast with the mesoporous MnO_2_, the typical O–H absorption bands at about 3000, 1620, 1081 and 589 cm^−1^ for the mesoporous MnO_2_/silver nanowires almost appear, moreover, the peaks at 2322, 2033 and 1641 cm^−1^ of the as-prepared silver nanowires belong to bending mode of hydroxyl groups and carbonyl groups, respectively, are still observed, suggesting that the addition of the silver nanowires modified the groups on the surface of the MnO_2_ [28].

### 2.3. Electrochemical Characterizations

As is well-known, silver is an excellent electronic conductor, and it has been used to dope electrode materials to improve their electrochemical performance in the past. The detail conductivity data of the samples are shown in Table 1. The chart shows that silver can change the electrical resistivity and electrical conductivity of mesoporous MnO_2_-4, and with an increase to the added amount of the silver, the electrical resistivity of the sample decreases and the electrical conductivity increases. Therefore, in the following tests of the electrical performance, a sample of MnO_2_:Ag = 1:1 was used.

To evaluate the potential applications as an electrode material for supercapacitors, the electrochemical properties of the mesoporous MnO_2_-based electrode material were investigated in a three-electrode system in 0.5 M Na_2_SO_4_ and firstly studied in 0.1 M, 0.2 M and 0.5 M KI electrolyte (Table 2). Some of the reported data beat the performance of the present paper, but KI electrolyte was studied in the first, and it may become a new area for supercapacitors to change the specific capacitance.

**Table 1:** The detail electrical resistivity (ER) and electrical conductivity (EC) of mesoporous MnO_2_ and manganese dioxide-based composite under different experimental conditions.

Sample	ER (Ω·cm)	EC (S/cm)
MnO_2_-4	2.54 × 10^6^	3.94 × 10^−7^
Ag	1.73 × 10^−2^	5.78 × 10
MnO_2_:Ag = 1:0.25	4.33 × 10^4^	2.31 × 10^−5^
MnO_2_:Ag = 1:0.5	1.10 × 10^2^	9.10 × 10^−3^
MnO_2_:Ag = 1:1	8.37	1.19 × 10^−1^

**Table 2:** Comparison of specific capacitances of the reported MnO_2_-based electrodes and the present work. All values are measured using the three-electrode system.

Samples	Cs (F·g^−1^)	Electrolyte	Test Condition	References
Ambigel MnO_2_	130	2 M NaCl	5 mV·s^−1^	[29]
α-MnO_2_ nanorod	152	1 M Na_2_SO_4_	5 mV·s^−1^	[30]
Birnessite hollow MnO_2_	169	1 M Na_2_SO_4_	0.25 A·g^−1^	[31]
MnO_2_ spherical particle	170.8	0.5 M K_2_SO_4_	0.5 A·g^−1^	[32]
MnO_2_ nanowire	176	1 M Na_2_SO_4_	5 mV·s^−1^	[33]
MnO_2_ nano hollow sphere	178	0.5 M K_2_SO_4_	0.5 A·g^−1^	[34]
Porous MnO_2_ nanoparticle	178.9	1 M Na_2_SO_4_	1 mV·s^−1^	[35]
MnO_2_ particle	180	0.5 M KOH	1 mV·s^−1^	[36]
Porous nano-MnO_2_	198.1	1 M Na_2_SO_4_	0.28 A·g^−1^	[37]
MnO_2_ nanoparticle	200	0.2 M K_2_SO_4_	5 mV·s^−1^	[38]
MnO_2_ nanowisker	200	1 M Na_2_SO_4_	2 mV·s^−1^	[39]
Amorphous MnO_2_·*n*H_2_O	200	2 M KCl	5 mV·s^−1^	[40]
MnO_2_ nanosheet array	201	1 M Na_2_SO_4_	1 A·g^−11^	[41]
Birnessite MnO_2_ nanosphere	210	1 M Na_2_SO_4_	1 A·g^−1^	[42]
Coral-like MnO_2_	221	1 M Na_2_SO_4_	0.5 A·g^−1^	[43]
MnO_2_ thin sheet	230	0.5 M Na_2_SO_4_	20 mV·s^−1^	[44]
γ-MnO_2_ film	240	0.1 M Na_2_SO_4_	1 mA·cm^−2^	[45]
Lamellar MnO_2_	242.1	2 M NH_4_(SO_4_)_2_	2 mA·cm^−2^	[46]
α-MnO_2_ nanorod	245	1 M KOH	1 A g^−1^	[47]
Amorphous MnO_2_ particle	251	1 M Na_2_SO_4_	2 mV·s^−1^	[48]
Mesoporous MnO_2_	200.3	0.5 M Na_2_SO_4_	0.1 A·g^−1^	This work
Mesoporous MnO_2_	205.2	0.5 M KI	0.1 A·g^−1^	This work
Mesoporous MnO_2_	200.6	0.5 M KI	0.2 A·g^−1^	This work
Mesoporous MnO_2_	197.1	0.5 M KI	0.5A·g^−1^	This work
Ag/Mesoporous MnO_2_	187.2	0.5 M Na_2_SO_4_	0.1 A·g^−1^	This work
Ag/Mesoporous MnO_2_	208.1	0.2 M KI	0.1 A·g^−1^	This work
Ag/Mesoporous MnO_2_	200.5	0.2 M KI	0.2 A·g^−1^	This work
Ag/Mesoporous MnO_2_	191.7	0.2 M KI	0.5 A·g^−1^	This work

**Figure 4:**
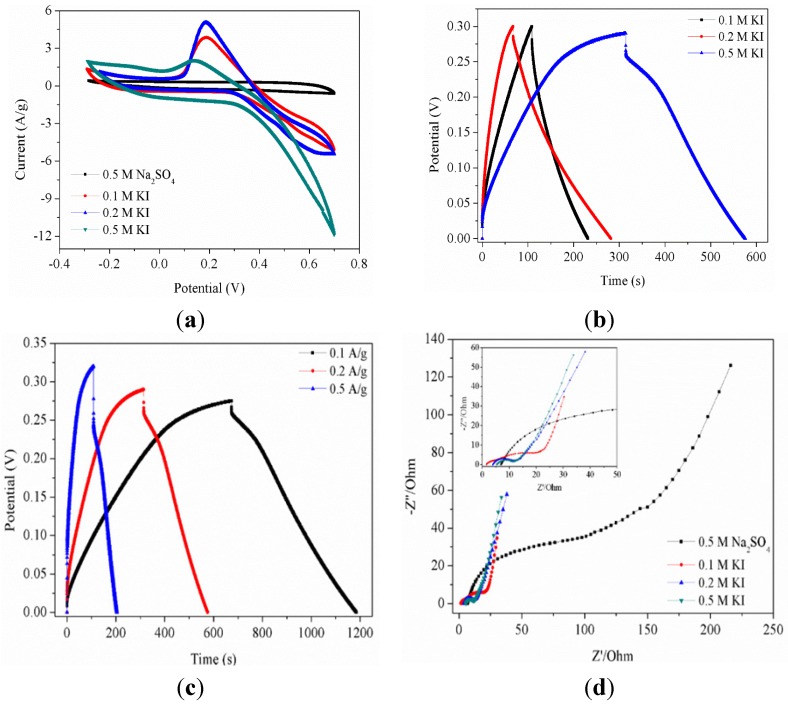
(**a**) cyclic voltammograms (CV) curves of mesoporous MnO_2_ at 5 mV/s in 0.5 M Na_2_SO_4_, 0.1 M, 0.2 M and 0.5 M KI electrolyte; (**b**) Galvanostatic charge/discharge curves of mesoporous MnO_2_ at 0.2 A/g in different molar concentration (0.1 M, 0.2 M and 0.5 M) KI electrolyte; (**c**) Galvanostatic charge/discharge curves of mesoporous MnO_2_ at 0.1, 0.2 and 0.5 A/g in 0.5 M Na_2_SO_4_ electrolyte; (**d**) Electrochemical impedance spectroscopy curves of mesoporous MnO_2_ in 0.5 M Na_2_SO_4_ and 0.1, 0.2 and 0.5 M KI electrolyte.

Figure 4a shows the CV curves of the mesoporous MnO_2_ electrode at a scan rate of 5 mV/s in different aqueous electrolytes (0.5 M Na_2_SO_4_, 0.1 M, 0.2 M and 0.5 M·KI) in the range of −0.3–0.7 V. It is clear that the CV curve of mesoporous MnO_2_ in 0.5 M Na_2_SO_4_ has no redox peaks over the potential range. The shape of the CV shows an approximate rectangular mirror image characteristic of capacitive behavior. This may be explained by the fact that mesoporous MnO_2_ occurred redox reaction in each potential. Therefore, there are generally no particularly prominent redox peaks in the CV curves. However, when the conductive of the electrode material is good and the scan rate is low, the cation can not only undertake the redox reaction on the surface, it is also possible carry out the redox reaction in the channels. In that case, obvious redox peaks will appear. Additionally, specific redox peaks have appeared in the CV curves of mesoporous manganese dioxide in KI electrolyte. The possible redox reaction of I^−^ at the electrode/electrolyte interface can be expressed as shown in Equation (1–4) [49,50,51]. These redox reactions may be the reasons that a higher specific capacitance of the electrode materials occurs KI than in Na_2_SO_4_ electrolyte.

3I^−^↔ I^−^_3_+ 2e^−^ (1)

2I^−^↔ I^−^_2_+ 2e^−^ (2)

2I^−^↔ 3I^−^_3_+ 2e^−^ (3)

I_2_+ 6H_2_O ↔ 2IO_3_^−^+ 12H^+^ + 10e^−^ (4)

Figure 4b presents the charge/discharge curves of mesoporous MnO_2_ at the experimental condition of 0.2 A/g in 0.1, 0.2 and 0.5 M KI electrolyte in the range of 0–0.30 V. It can be seen that with the increase of the concentration of I^−^, the capacitance of the mesoporous MnO_2_ electrode also increases. All of the specific capacitance of the mesoporous MnO_2_ in the KI electrolyte are higher than those in the Na_2_SO_4_ electrolyte. Figure 4c shows the charge/discharge curves of the mesoporous MnO_2_ electrode measured at different current densities (0.1, 0.2 and 0.5 A/g) in 0.5 M KI electrolyte. From the detailed data shown in Table 3, it is clear that the mesoporous MnO_2_ electrode material has a high rate of specific capacitance in 0.5 M KI electrolyte. Electrochemical impedance spectroscopy (EIS) measurements were taken to study the storage kinetics of all of the as-prepared materials. As shown in Figure 4d, the EIS of all samples was composed of a partially overlapped semicircle and a straight sloping line. Additionally, the diameter of the semicircle is related to the resistance of the electrode materials. As the diameter decreased, so did the resistance. It was found that the resistance of the electrode materials was the smallest in the 0.5 M KI electrolyte. This is related to the previous discussion.

**Figure 5:**
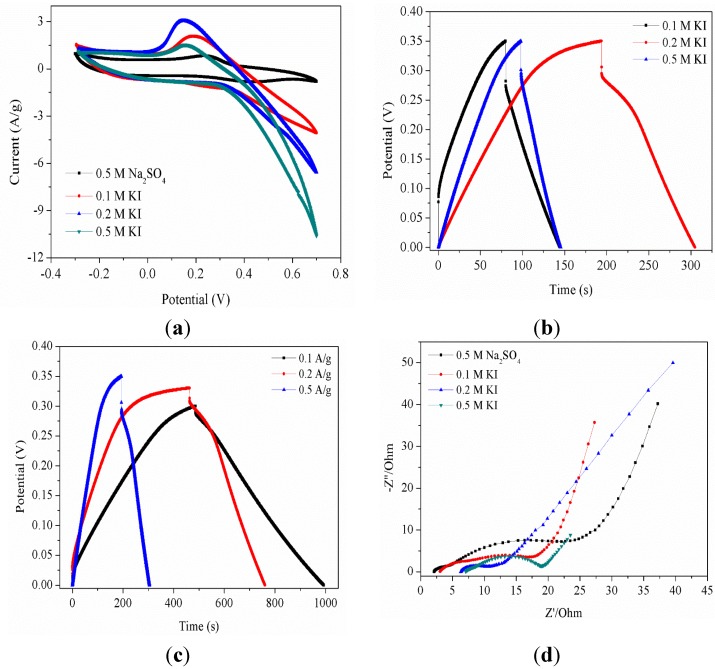
(**a**) CV curves of mesoporous MnO_2_/silver nanowires at 5 mV/s in 0.5 M Na_2_SO_4_, 0.1 M, 0.2 M and 0.5 M KI electrolyte; (**b**) Galvanostatic charge/discharge curves of mesoporous MnO_2_/silver nanowires at 0.2 A/g in different molar concentration (0.1 M, 0.2 M and 0.5 M) KI electrolyte; (**c**) Galvanostatic charge/discharge curves of mesoporous MnO_2_/silver nanowires at 0.1, 0.2 and 0.5 A/g in 0.5 M Na_2_SO_4_ electrolyte; (**d**) Electrochemical impedance spectroscopy curves of mesoporous MnO_2_/silver nanowires in 0.5 M Na_2_SO_4_ and 0.1, 0.2 and 0.5 M KI electrolyte.

Figure 5a shows the CV curves of the mesoporous MnO_2_/silver nanowires electrodes at a scan rate of 5 mV/s in different aqueous electrolytes (0.5 M Na_2_SO_4_, 0.1 M, 0.2 M and 0.5 M KI) in the range of −0.3–0.7 V. It can be seen that the CV curve of the mesoporous MnO_2_/silver nanowires in 0.5 M Na_2_SO_4_ has redox peaks appeared over the potential range. However, the area of the CV curve of the mesoporous MnO_2_/silver nanowires in 0.5 M Na_2_SO_4_ was obviously smaller than that of the other CV curves, which reflects the fact that the specific capacitance of the mesoporous MnO_2_/silver nanowires in 0.5 M Na_2_SO_4_ electrolyte was smaller than the specific capacitance of the mesoporous MnO_2_/silver nanowires in KI electrolyte. Figure 5b presents the charge/discharge curves of the mesoporous MnO_2_/silver nanowires composite electrode at the experimental condition of 0.5 A/g in 0.1, 0.2 and 0.5 M KI electrolyte in the range of 0–0.35 V. For the pure MnO_2_, the large Warburg region indicates great variation in the ion diffusion path lengths and an increase of ion movement obstruction in the process of ion diffusion into the interior of the electrode. This implies that silver nanowire dopant in MnO_2_ can promote the ion diffusion rate, which explains the fast current response in the CV curve and the ideal capacitive behavior of the mesoporous MnO_2_/silver nanowires composite electrode. Obviously, the charge/discharge curve results confirm that the silver nanowires dopant in MnO_2_ not only enhances the electron transfer efficiency but also facilitates ion diffusion [52,53,54]. Additionally, due to the synergistic effect of the electrical conductivity of the electrode and the electrolyte, the specific capacitance of the mesoporous MnO_2_/silver nanowires reaches its maximum in 0.2 M KI electrolyte. Figure 5c presents the charge/discharge curves of the mesoporous MnO_2_/silver nanowires composite electrode with different current densities in 0.2 M KI in the range of 0–0.35 V. It is clear that with an increase of the current density, the charge/discharge voltage also increases, and a larger voltage drop appeared. These results imply that the changing test conditions also affected the concentration of iodine ions in the electrode surface and the electrolyte. As shown in Figure 5d, the EIS of all of the samples was composed of a partially overlapped semicircle and a straight sloping line. It is noted that for all samples, at very high frequency, the intercepts at real part Z’ were almost the same, indicating the same combination resistance of ionic resistance of electrolyte, intrinsic resistance of active materials and contact resistance at the active material/current collector interface [55,56].

All of the relevant specific capacitances (*C_s_*) were calculated from the galvanostatic charge/discharge according to Equation (5) [57,58,59], and all of the details of the data are shown in Table 3 and Table 4.

(5) $$C_{S}=\frac{i\times \Delta t}{m\times \Delta V}$$

where *C_s_* is the specific capacitance (F/g), *i* is the discharge current (A), ∆*t* is discharge time (s), *m* is the active mass of electrode (g), and ∆*V* is the total potential deviation (V).

It can be observed that the manganese dioxide-based nanocomposite materials exhibited the highest specific capacitance, and the specific capacitance of the mesoporous MnO_2_ that was tested in KI electrolyte was higher than that which was in Na_2_SO_4_ electrolyte. Besides, with the increase of the concentration of I^−^, the specific capacitance of the mesoporous MnO_2_ electrode material also increased accordingly. In addition, the mesoporous MnO_2_ showed a good ratio discharge property in 0.5 M KI electrolyte. In fact, the specific capacitance reached 205.5, 200.6 and 197.1 F/g at 0.1, 0.2 and 0.5 A/g, respectively. This may be because of the effect of the electrical conductivity of the electrolyte. On the other hand, the specific capacitance of mesoporous MnO_2_ also changed when the blended with silver nanowires. However, according to the detailed data as shown in Table 4, the specific capacitance of mesoporous MnO_2_/silver nanowires reached its maximum in 0.2 M KI electrolyte. This may be due to the synergistic effect of the electrical conductivity of the electrode and the electrolyte, the conductivity of the composite electrode and the ion activity of the electrolyte were worked together to the electrode materials, make sure that it has a good ratio discharge property under different test conditions [60,61]. Finally, the mesoporous MnO_2_/silver nanowires show a good ratio discharge property in 0.2 M KI electrolyte, with the specific capacitance reaching to 208.1, 200.5 and 191.7 F/g at 0.1, 0.2 and 0.5 A/g, respectively.

**Table 3:** The detail specific capacitance (F/g) of mesoporous MnO_2_ under different experimental conditions.

Sample	Mesoporous MnO_2_			
Experimental Conditions	0.1 M KI	0.2 M KI	0.5 M KI	0.5 M Na_2_SO_4_
0.1 A/g	106.6	229.2	205.5	200.3
0.2 A/g	89.3	152.5	200.6	85.72
0.5 A/g	57.1	152.9	197.1	49.12

**Table 4:** The detail specific capacitance (F/g) of mesoporous MnO_2_/silver nanowires under different experimental conditions.

Sample	Mesoporous MnO_2_/Silver Nanowires			
Experimental Conditions	0.1 M KI	0.2 M KI	0.5 M KI	0.5 M Na_2_SO_4_
0.1 A/g	152.1	208.1	158.1	187.2
0.2 A/g	130.8	200.5	133.7	150.2
0.5 A/g	117.2	191.7	115.8	113.1

## 3. Experimental Section

### 3.1. Preparation of Mesoporous MnO_2_

First, 1.730 g KMnO_4_ was dissolved in 100 mL of deionized water and stirred with a magnetic stirrer for 30 min to form a homogeneous solution at room temperature. Then, 2 mL concentrated sulfuric acid were added in 2.5 mL of ethylene glycol and aged for 20 min with stirring at ambient conditions. Then the mixture was poured into the above KMnO_4_ solution. The mixture was aged for 20 min with stirring at ambient conditions. The deposits were collected by centrifugation, washed several times with deionized water and absolute ethanol until the pH of the wash was neutral, and then dried at 60 °C overnight under air atmosphere. This sample was denoted as MnO_2_-2 for the following tests [25]. In the remaining three experiments, 4, 5 and 7 mL concentrated sulfuric acid were added in the ethylene glycol solution, respectively, and the collected samples were correspondingly denoted as MnO_2_-4, MnO_2_-5 and MnO_2_-7 for the following tests.

### 3.2. Preparation of Silver Nanowires

First, 5 mL of ethylene glycol was added to a disposable glass vial to which a stir bar was added; the vial was then suspended in an oil bath at 151.5 °C and heated for 1 h under magnetic stirring. At the same time, reagent solutions were prepared. At 1 h, a Cu-additive solution was injected into the heated ethylene glycol. The solution was then heated for an additional 15 min. 1.5 mL of a 0.147 M polyvinylpyrrolidone solution in ethylene glycol, followed by 1.5 mL of a 0.094 M AgNO_3_ solution in ethylene glycol [26,62]. Then, the reaction was quenched by cooling the reaction vial in a room temperature water bath. Products were then washed with acetone and water.

### 3.3. Preparation of Mesoporous Manganese Dioxide-Based Composite Materials

Silver nanowires and mesoporous MnO_2_ were mixed directly with different molar ratios, and to reach the uniform by grinding.

### 3.4. Material Characterization

The Nitrogen adsorption-desorption isotherms were tested with a JW-BK Static Physisorption Analyzer. The BET surface area was calculated from the desorption branches in the relative pressure range of 0.05–0.35, and the total pore volume and average pore diameter were evaluated at a relative pressure of about 0.99. The materials were pretreated at 300 °C for 6 h. Pore-size distributions (PSD) were calculated by Barrett-Joyner-Halenda (BJH) plots using the nitrogen desorption isotherm. The pore width was obtained at the peak of the PSD curve. The morphologies of the prepared products were examined by scanning electron microscopy (SEM), using a FEI Quanta 400 F at 20 kV. X-ray diffraction (XRD) patterns were obtained with a Bruker D8 Diffractometer in reflection mode using Cu Kα = 0.154 nm with a voltage of 40 kV. In addition, Fourier transform infrared spectrometry (FT-IR) analyses were performed on a Thermal Nicolet Infrared Spectrometer.

### 3.5. Preparation of Electrodes and Electrochemical Characterization

The working electrodes were fabricated by the as-prepared materials, carbon black and poly tetrafluoroethylene (PTFE) at a weight ratio of 70:20:10. Typically, the mixture was formed to slurry by adding a few drops of 1-methyl-2-pyrrolidinone, then coated onto a stainless steel grid with an apparent area of 1 cm × 1 cm, and finally dried under air atmosphere at 70 °C for 2 h. All electrochemical measurements were done in a three-electrode system. The prepared electrodes were impregnated with different aqueous electrolytes before the electrochemical tests for 0.5 h at room temperature. A Pt wire and an Ag/AgCl cell were used as counter and reference electrodes, respectively. The CV and galvanostatic charge/discharge (GC) were measured by a CHI 660C electrochemical workstation. CV tests were performed between −0.3 and 0.7 V. Galvanostatic charge/discharge curves were measured in the potential range of −0.3–0.7 V at a current density of 0.5 A/g.

The conductivity tests were carried out according to Four Probes Tech type RTS-9. The ambient temperature of the experiments was in the range 23 ± 5 °C and the operating humidity was in the range 73 ± 5 °C. The samples were prepared with a typical diameter of around 13 mm and thick layer of around 0.5 mm by a tablet machine.

## 4. Conclusions

In this study, uniform mesoporous MnO_2_ was obtained through a simple one-pot synthesis procedure at ambient temperature from KMnO_4_ and ethylene glycol under acidic conditions. Subsequently, the electrochemical performance of the resulting mesoporous MnO_2_-based electrode materials was tested in KI and Na_2_SO_4_ electrolytes. The mesoporous MnO_2_ showed a good ratio discharge properties in 0.5 M KI electrolyte, the strength of the current was amplified 5 times (from 0.1 to 0.5 A/g), and the remain rate of the specific capacitance was 95% (from 205.5 down to 197.1 F/g). Due to the synergistic effect of the electrical conductivity of the electrode and the electrolyte, the mesoporous MnO_2_/silver nanowires showed a good ratio discharge property in 0.2 M KI electrolyte, with the strength of the current being amplified five times (from 0.1 to 0.5 A/g), and the remain rate of specific capacitance at 92% (from 208.1 down to 191.7 F/g). The rate performance was also much higher than that in Na_2_SO_4_ electrolyte, where the remain rate were 25% (from 200.3 down to 49.1 F/g) and 60% (from 187.2 down to 113.1 F/g), respectively. Results indicate that KI electrolyte may become a new way for supercapacitors to change the specific capacitance.
